# Multimodal data-driven predictive model for vancomycin serum concentrations in postoperative cardiac surgery patients under cardiopulmonary bypass: a single-center retrospective study

**DOI:** 10.3389/fphar.2025.1707557

**Published:** 2026-01-08

**Authors:** Pengqiang Du, Wenjin Zhang, Yujie Zhou, Taoran Li, Xinlei Qi, Wei Si, Aifeng Wang, Yongcheng Ma, Xingang Li

**Affiliations:** 1 Henan Key Laboratory of Individualized Drug Therapy for Cardiovascular Diseases, Department of Pharmacy, Fuwai Central China Cardiovascular Hospital, Central China Fuwai Hospital of Zhengzhou University, Zhengzhou, China; 2 National Health Commission Key Laboratory of Cardiovascular Regenerative Medicine, Central China Subcenter of National Center for Cardiovascular Diseases, Henan Cardiovascular Disease Center, Fuwai Central China Cardiovascular Hospital, Central China Fuwai Hospital of Zhengzhou University, Zhengzhou, China; 3 Department, Central-China Branch of National Center for Cardiovascular Diseases, Henan Cardiovascular Disease Center, Fuwai Central China Cardiovascular Hospital, Central China Fuwai Hospital of Zhengzhou University, Zhengzhou, China; 4 Department of Pharmacy, Beijing Friendship Hospital, Capital Medical University, Beijing, China

**Keywords:** cardiopulmonary bypass, LASSO regression, plasma concentration, post-cardiac surgery, predictive model, vancomycin

## Abstract

**Background:**

This study analyzed the pharmacokinetic data of vancomycin in patients after cardiac surgery under cardiopulmonary bypass (CPB). It aimed to identify factors affecting vancomycin plasma concentrations and develop a multi-modal data-driven prediction model for these concentrations.

**Methods:**

A retrospective study included hospitalized patients who underwent cardiac surgery under cardiopulmonary bypass, received intravenous vancomycin for postoperative infection, and had plasma concentration monitoring. Patient demographic and other relevant information was collected. A dual-risk model based on the therapeutic window (10–20 mg/L) was established: ① nephrotoxicity risk group (≥20 mg/L vs. < 20 mg/L, n = 350); ② subtherapeutic concentration risk group (<10 mg/L vs. 10–20 mg/L, n = 211). Missing data were imputed using the K-nearest neighbor algorithm (KNN, K = 10) to ensure data integrity. Univariate logistic regression (α = 0.10) was used for initial variable selection. LASSO regression with 10-fold cross-validation selected optimal features, and a multivariate bidirectional stepwise regression (forward-backward method, AIC criterion) built the prediction model. Model validation used forest plots, nomograms, ROC curves, calibration curves, and decision curve analysis (DCA). Generalization performance was assessed via ten-fold cross-validation.

**Results:**

Our models demonstrated strong predictive performance for both nephrotoxicity and subtherapeutic concentration risks. In the nephrotoxicity model, creatinine clearance rate (CCr) (OR = 0.975, 95% CI: 0.965–0.984; P < 0.001) and CPB duration (OR = 1.200, 95% CI: 1.068–1.350; P = 0.002) were identified as independent predictors, achieving an AUC of 0.733 (95% CI: 0.680–0.787). For the subtherapeutic concentration risk model, age (OR = 1.035, 95% CI: 1.007–1.064; P = 0.016) and estimated glomerular filtration rate (eGFR) (OR = 0.981, 95% CI: 0.964–0.998; P = 0.032) were significant predictors, with an AUC of 0.758 (95% CI: 0.692–0.824). Both models showed good calibration (P > 0.05) on the Hosmer-Lemeshow test, with better discrimination in the low concentration risk group. DCA confirmed superior clinical net benefit for both models over full/intervention strategies. In ten-fold cross-validation, the AUC fluctuation was <5%, indicating good stability.

**Conclusion:**

These models effectively predict steady-state vancomycin concentrations in post-CPB patients. Lower CCr and longer CPB duration increase nephrotoxicity risk, while younger age and higher eGFR were associated with increased subtherapeutic concentration risk. These findings facilitate early identification of high-risk patients by clinicians, enabling timely intervention for optimized dosing regimens.

## 1 Introduction

Cardiac surgery patients are at high risk for drug-resistant Gram-positive bacterial infections due to factors such as cardiopulmonary bypass (CPB), valve replacement, multiple indwelling catheters, and infective endocarditis. Studies show that nosocomial infections in these patients primarily manifest as respiratory tract infections, central venous catheter-related infections, and surgical site infections, predominantly caused by Gram-positive bacteria, particularly *Staphylococcus* epidermidis and *Staphylococcus aureus* (Michalopoulos et.al., 2006). Vancomycin, a representative glycopeptide antibiotic, remains the first-line treatment for methicillin-resistant *Staphylococcus aureus* (MRSA) infections due to its broad-spectrum activity against Gram-positive bacteria (Liu et al., 2011). However, vancomycin has a narrow therapeutic window. Subtherapeutic serum concentrations increase the risk of treatment failure and resistance development, while supratherapeutic levels elevate nephrotoxicity risk. This challenge is particularly pronounced in post-CPB patients. Research indicates that individualized dosing guided by clinical decision support systems can improve trough concentration target attainment, enhance clinical efficacy, and reduce nephrotoxicity (Bassetti et al., 2022).

Notably, guidelines prioritize the area under the concentration-time curve (AUC) over trough concentration (Ctrough) as the optimal target for vancomycin TDM, as AUC more comprehensively reflects total drug exposure and correlates better with efficacy (AUC_0-24_h 400–600 mg h/L for mild-moderate infections) (Rybak et al., 2020b). However, in the clinical context of post-CPB cardiac surgery, Ctrough monitoring remains the most feasible option due to its simplicity and rapid availability: postoperative patients with infections require timely dosing regimens, while AUC calculation typically demands multiple blood samples (e.g., peak and trough concentrations) or population pharmacokinetic modeling, which is logistically challenging in the acute postoperative phase. In parallel, the updated Chinese Vancomycin TDM Guidelines (2020) issued by the Chinese Pharmacological Society recommend using TDM to maintain trough concentrations within the range of 10–20 mg L^-1^ (He et al., 2020).

Nevertheless, in post-cardiac surgery patients, trough concentrations <10 mg L^-1^ are associated with suboptimal efficacy and increased treatment failure risk, while levels >20 mg L^-1^ heighten the risk of adverse events like nephrotoxicity. This is especially critical for patients with underlying risk factors such as cardiorenal syndrome or postoperative low cardiac output syndrome (Wang et al., 2023). Currently, most research focuses on general patient populations, with limited specific analysis for post-CPB cardiac surgery patients. Studies on vancomycin in CPB patients often concentrate on intraoperative pharmacokinetics (Cotogni et al., 2013; Miglioli et al., 1998), yielding conflicting results: one found no significant impact of CPB on intraoperative vancomycin PK, while another reported decreased serum levels during CPB.

This retrospective study addresses this gap by developing a prognostic prediction model using multimodal data to stratify vancomycin concentration-related risks in post-CPB cardiac patients. Model validity was rigorously evaluated to support personalized dosing strategies, ultimately aiming to optimize therapeutic outcomes while minimizing toxicity.

## 2 Methods

### 2.1 Study design and ethics approval

This single-center retrospective study included patients who underwent cardiac surgery with CPB at Fuwai Central China Cardiovascular Hospital between January 2020 and December 2023 and received vancomycin for postoperative infections. The study protocol was approved by the Fuwai Central China Cardiovascular Hospital Ethics Committee (Approval No. 2023-79), which granted a waiver of informed consent for this retrospective analysis. Trial registration: This trial was registered at ClinicalTrials.gov under identifier NCT07087301 on 17 July 2025 (retrospectively registered). All medical records and biological specimens were handled in compliance with relevant regulations and ethical principles.

### 2.2 Patient inclusion

Patient medical records were reviewed to extract the following clinical data: age, sex, weight, height, CPB duration, surgery duration, concomitant antibiotics, vancomycin dosing regimen, serum creatinine (SCr), serum albumin, blood urea nitrogen (BUN), estimated glomerular filtration rate (eGFR), vancomycin trough serum concentration, comorbidities, and smoking/alcohol history. Vancomycin trough concentrations were measured using enzyme-multiplied immunoassay technique (EMIT). The estimation of eGFR was performed using the CKD-EPI CYS-C equation (Choi et al., 2024). Creatinine clearance (CCr) was calculated using the Cockcroft-Gault formula (Cucci et al., 2023).

Patients meeting any of the following criteria were excluded: (i) age <18 years; (ii) missing data on weight, height, or serum creatinine during vancomycin therapy; (iii) absence of vancomycin trough plasma concentration measurements.

### 2.3 Data collection and evaluation

In this study, a strict data quality control process was used to ensure the accuracy and reliability of the analyzed data. This included establishing unified data collection standards covering patient demographics, lab test results, etc., using a secure centralized database with validation checks for data entry, and ensuring data integrity and accuracy via double data entry and cross - verification. Moreover, regular data quality audits were conducted to check data completeness and adherence to the study protocol, making sure data collectors and the research team strictly followed data quality standards throughout. These measures aimed to enhance data robustness and validity, thereby strengthening the credibility of the study conclusions.

### 2.4 Predictive model performance

A systematic data processing and modeling approach was used to develop vancomycin plasma concentration prediction models. Missing data were multiply imputed using the K-nearest neighbor algorithm (KNN, K = 10), which calculates weighted imputation values based on Euclidean distances in feature space to preserve original data distribution. To address multicollinearity, variance inflation factor (VIF) testing was performed on all candidate predictors, excluding variables with VIF >5. A dual-model framework was established based on vancomycin’s therapeutic window (10–20 mg/L): the first model (for nephrotoxicity risk prediction) defined high concentration as ≥20 mg/L and control as <20 mg/L to identify risk factors for vancomycin accumulation. The second model (for subtherapeutic concentration risk prediction) defined low concentration as trough <10 mg/L and target as 10–20 mg/L to analyze predictors of target concentration attainment.

### 2.5 Feature selection and model development

Both models used the same stepwise variable selection process. Initially, univariate logistic regression was done on all candidate variables, keeping those with P-values <0.1 after false discovery rate (FDR) correction for further analysis. LASSO regression with 10-fold cross-validation was applied to identify variables with non-zero coefficients and select the most explanatory predictor combinations. The optimal penalty coefficient (λ) was selected based on the criterion that minimizes the cross-validation error (lambda.min), as this approach prioritizes the model’s predictive performance. Then, two-directional stepwise regression (forward - backward) based on the Akaike information criterion (AIC) was implemented to build the prediction model. The AIC-based approach optimizes the overall model fit rather than enforcing individual predictor significance, which means that variables selected by LASSO might be excluded in this final step if their information is captured by a stronger correlate, or conversely, retained due to their contribution to the model’s parsimony even if their p-value is not statistically significant. This approach reduces overfitting and captures clinically valuable predictors (Frank and Harrell, 2015). The final model built through this layered screening (statistical significance, regularization-based dimensionality reduction, model - driven optimization) ensures interpretability and robustness.

### 2.6 Model validation and evaluation

The study comprehensively evaluated the nephrotoxicity and subtherapeutic concentration risk prediction models via the same multidimensional system. First, using receiver operating characteristic (ROC) curves, we calculated area under the curve (AUC) and 95% confidence intervals to assess model discrimination. Hosmer-Lemeshow tests and calibration curves visualized the consistency between predicted probabilities and actual observations. For clinical utility quantification, decision curve analysis (DCA) measured net benefits at different risk thresholds. To rigorously assess model stability and potential overfitting, an internal validation was performed using the bootstrap method with 1,000 resamples. The optimism-corrected performance metrics, including the bootstrap-validated AUC, were calculated. Model stability was tested via 10-fold cross-validation, with AUC fluctuations reflecting generalization ability. Nomograms visually presented each predictor’s weight and clinical value, while forest plots showed effect sizes and confidence intervals, offering visual interpretation of the prediction mechanism.

### 2.7 Statistical analysis

All data analyses were performed using R 4.3.2. Key analysis modules used VIM for KNN imputation, car for VIF analysis, glmnet for LASSO regression, MASS for stepwise regression,rms for nomogram building, pROC for ROC curve plotting, rmda for DCA, forestplot for forest plot generation, gplots for Hosmer-Lemeshow testing and ggplot2 for calibration curves. Univariate analysis was conducted using generalized linear models (glm) from the stats package. During model development, univariate screening used a lenient P < 0.1 threshold to retain potential predictors. LASSO regression selected the optimal variable set via 10-fold cross-validation (lambda.min). Stepwise regression optimized variables based on the AIC criterion without enforcing predictor significance.

For data presentation, categorical variables are summarized as frequencies (percentages). Continuous variables were assessed for normality using the Shapiro-Wilk test from the stats package, with non-normally distributed data described as median (interquartile range) and normally distributed data expressed as mean ± standard deviation. For group comparisons, Mann-Whitney U tests (non-normal) or independent samples t-tests (normal) were used for continuous variables, and χ^2^ or Fisher’s exact tests for categorical variables. All statistical tests were two-tailed, with significance at α = 0.05.

## 3 Results

### 3.1 Basic characteristics of research participants

This study included 350 participants, comprising 233 males (66.6%) and 117 females (33.4%), with a mean age of 56.57 ± 12.46 years. Table 1 presents the demographics and clinical characteristics of all patients. For the nephrotoxicity risk prediction model, patients were categorized into two groups based on their vancomycin steady-state serum concentration: the high-risk group (≥20 mg/L, n = 139, mean age 57.22 ± 12.23 years) and the low-risk group (<20 mg/L, n = 211, mean age 56.15 ± 12.61 years). Significant differences were found between these two groups in BUN, SCr, CCr, eGFR, vancomycin dosage, and CPB duration (p < 0.05). In the subtherapeutic concentration risk prediction model, patients were divided into the target group (serum concentration of 10–20 mg/L, n = 136, mean age 59.10 ± 11.46 years) and the low-concentration risk group (<10 mg/L, n = 75, mean age 50.81 ± 12.92 years). Significant differences existed between these two groups in age, BUN, SCr, eGFR, CCr, and surgical duration (p < 0.05). Moreover, variance inflation factor tests on the grouped data for both the nephrotoxicity risk and subtherapeutic concentration risk prediction models showed no multicollinearity among the variables included in the analyses.

**TABLE 1:** Comparison of demographic and clinical characteristics between patients in the kidney injury risk group and the low-concentration risk group.

Characteristic	Overall (n = 350)	Nephrotoxicity		p value	VIF	Therapeutic effect		p value	VIF
		High-risk group (n = 139)	Low-risk group (n = 211)			Target group (n = 136)	Failure risk group (n = 75)		
Individual characteristics, mean ± SD									
Age	56.57 ± 12.46	57.22 ± 12.23	56.15 ± 12.61	0.435	1.28	59.10 ± 11.46	50.81 ± 12.92	<0.001	1.32
BMI	24.75 ± 3.47	24.45 ± 3.28	24.95 ± 3.58	0.180	1.22	24.67 ± 3.32	25.47 ± 3.99	0.139	1.25
Demographics (Samples%)									
Gender	​	​	​	0.056	1.39	​	​	0.112	1.39
Female	117 (33)	55 (39.57)	63 (29.72)	​	​	45 (33.1)	17 (12.5)	​	​
Male	233 (67)	84 (60.43)	149 (70.28)	​	​	91 (66.9)	58 (42.6)	​	​
Surgical duration and renal laboratory metrics, median[IQR]									
ALB	43.4 [40.58–47]	43.7 [40.3–47.9]	43.1 [40.7–46.55]	0.451	1.02	43.1 [40.25–46.48]	43.2 [41.4–47.4]	0.188	1.04
BUN	10.25 [7.6–13.43]	11.6 [8.9–16]	9.4 [7.2–12.05]	<0.001	1.18	9.6 [7.5–13.08]	8.6 [6.3–11.6]	0.03	1.11
SCr	93.5 [76–122.25]	111 [84–148]	88 [73–107]	<0.001	1.79	92 [76–113.5]	82 [71–94]	0.008	1.69
Ccr	68.59 [50.66–88.79]	56.38 [41.34–70.27]	78.12 [59.75–94.29]	<0.001	1.89	68.93 [53.61–88.14]	89.95 [77.24–107.3]	<0.001	1.81
eGFR	65.04 [50.71–81.55]	57.82 [48.58–73.24]	71.58 [53.42–87.15]	<0.001	1.53	64.21 [48.78–81.5]	81.33 [68.74–92.04]	<0.001	1.55
CPB duration	3.31 [2.5–4.8]	4 [2.63–5.28]	3 [2.3–4.36]	<0.001	1.11	3.1 [2.3–4.69]	2.85 [2.18–3.83]	0.080	1.14
Surgery duration	6 [4.5–7.6]	6.4 [4.5–8.17]	5.8 [4.5–7.33]	0.215	1.11	5.5 [4.3–7]	6.08 [5–7.5]	0.043	1.17
Pharmacotherapy (samples%)									
Vancomycin dosage and administration		​	​	0.038	1.06	​	​	0.13	1.09
Vancomycin usage 1 (1 g q12h)	146 (42)	64 (46.04)	82 (38.86)	​	​	51 (37.5)	31 (41.3)	​	​
Vancomycin usage 2 (0.5 g q6h)	52 (15)	13 (9.35)	39 (18.48)	​	​	20 (14.7)	19 (25.3)	​	​
Vancomycin usage 3 (0.5 g q8h)	112 (32)	50 (35.97)	62 (29.38)	​	​	44 (32.4)	18 (24.0)	​	​
Vancomycin usage 4 (0.5 g q12h)	40 (11)	12 (8.63)	28 (13.27)	​	​	21 (15.4)	7 (9.3)	​	​
Combined use of vasoactive drugs		​	​	0.699	1.16	​	​	0.125	1.19
Yes	283 (81)	111 (79.86)	172 (81.52)	​	​	115 (84.6)	57 (76.0)	​	​
No	67 (19)	28 (20.14)	39 (18.48)	​	​	21 (15.4)	18 (24.0)	​	​
Combined use of antibiotics		​	​	0.953	1.13	​	​	0.242	1.14
Yes	327 (93)	130 (93.53)	197 (93.36)	​	​	129 (94.9)	68 (90.7)	​	​
No	23 (7)	9 (6.47)	14 (6.64)	​	​	7 (5.1)	7 (9.3)	​	​
Comorbidities and lifestyle (samples%)									
Diabetes	​	​	​	0.463	1.06	​	​	0.89	1.09
Yes	46 (13)	16 (11.51)	30 (14.22)	​	​	19 (14.0)	11 (14.7)	​	​
No	304 (87)	123 (88.49)	181 (85.78)	​	​	117 (86.0)	64 (85.3)	​	​
Hyperlipemia	​	​	​	0.689	1.06	​	​	0.662	1.08
Yes	35 (10)	15 (10.79)	20 (9.48)	​	​	12 (8.8)	8 (10.7)	​	​
No	315 (90)	124 (89.21)	191 (90.52)	​	​	124 (91.2)	67 (89.3)	​	​
Hypertension	​	​	​	0.349	1.09	​	​	0.071	1.11
Yes	138 (39)	59 (42.45)	79 (37.44)	​	​	57 (41.9)	22 (29.3)	​	​
No	212 (61)	80 (57.55)	132 (62.56)	​	​	79 (58.1)	53 (70.7)	​	​
Alcohol	​	​	​	0.141	1.27	​	​	0.137	1.27
Yes	101 (29)	34 (24.46)	67 (31.75)	​	​	48 (35.3)	19 (25.3)	​	​
No	249 (71)	105 (75.54)	144 (68.25)	​	​	88 (64.7)	56 (74.7)	​	​
Smoke	​	​	​	0.032	1.39	​	​	0.072	1.41
Yes	153 (44)	51 (36.69)	102 (48.34)	​	​	72 (52.9)	30 (40.0)	​	​
No	197 (56)	88 (63.31)	109 (51.66)	​	​	64 (47.1)	45 (60.0)	​	​

BMI, body mass index; ALB, albumin; BUN, blood urea nitrogen; SCr, Serum Creatinine; Ccr, creatinine clearance; eGFR, estimated glomerular filtration rate; CPB, cardiopulmonary bypass.

### 3.2 Univariate analysis for selecting preliminary risk factors

**FIGURE 1:**
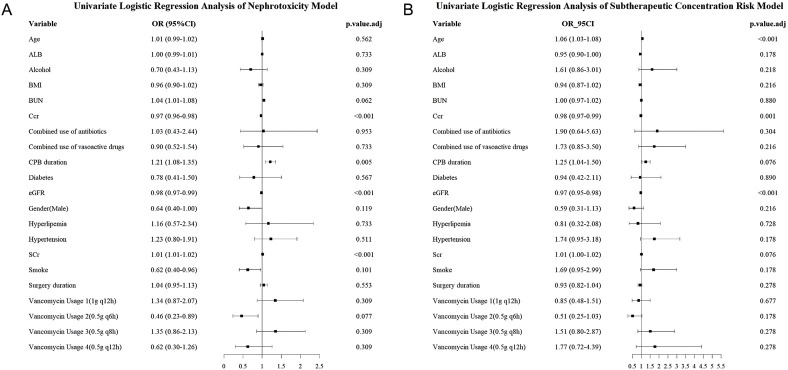
Forest plot illustrating effect estimates of variables from univariate regression analysis. **(A)** Nephrotoxicity model, **(B)** Subtherapeutic concentration risk model.

Variables with p < 0.1 in univariate analysis were retained for further selection. Key predictors included creatinine clearance (CCr), estimated glomerular filtration rate (eGFR), age, and cardiopulmonary bypass (CPB) duration, consistent with established pharmacokinetic influences in post-CPB patients. Figure 1 presents forest plots showing the effect estimates of each variable from the univariate regression analyses for both risk groups.

### 3.3 LASSO-logistic regression for screening feature variables

**FIGURE 2:**
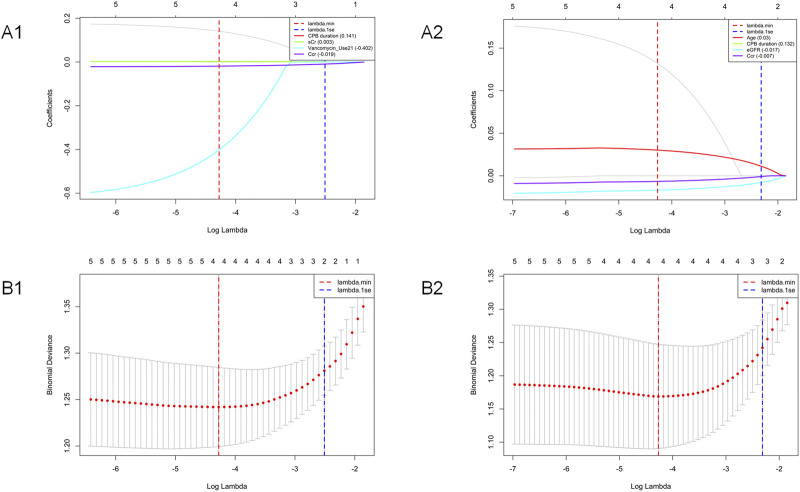
Variable selection using LASSO regression with 10-fold cross-validation. **(A1)** Cross-validation error curves for the nephrotoxicity risk model. **(A2)** Cross-validation error curves for the subtherapeutic concentration risk model. The red and blue dashed vertical lines indicate the positions of lambda. min and lambda.1se, respectively. **(B1)** Coefficient paths showing variable shrinkage for the nephrotoxicity risk model. **(B2)** Coefficient paths showing variable shrinkage for the subtherapeutic concentration risk model. The vertical lines correspond to the lambda. min (red) and lambda.1se (blue) positions. Variables with coefficients shrinking to zero are excluded from the final model.

Based on the variables screened by univariate analysis, this study further employed LASSO regression to identify independent risk factors associated with vancomycin plasma concentrations. As the regularization parameter λ increased, the regression coefficients of all variables gradually approached zero, and the number of variables retaining non-zero coefficients correspondingly decreased, as visualized in the coefficient paths (Figures 2A1, A2) The optimal λ value was determined using 10-fold cross-validation. The primary criterion was lambda. min (λ = 0.0139 for the nephrotoxicity model and λ = 0.0140 for the subtherapeutic model), which minimizes the cross-validation error (Figures 2B1, B2). This criterion was selected to optimize the model’s predictive performance by retaining variables that contribute most significantly to prediction accuracy. For comparison, the lambda.1se criterion, which yields a more parsimonious model within one standard error of the minimum error, was also examined but resulted in a marginally higher cross-validation error. Ultimately, the renal injury risk prediction model selected 4 predictor variables with non-zero coefficients: CPB duration, SCr level, vancomycin regimen 2 (0.5 g q6h), and CCr. The low-concentration risk prediction model selected 4 predictor variables: age, CPB duration, eGFR, and CCr. Both models effectively reduced dimensionality through LASSO regression, providing key predictor variables for subsequent multivariate analysis.

### 3.4 Multivariate stepwise regression for identifying key predictors

**FIGURE 3:**
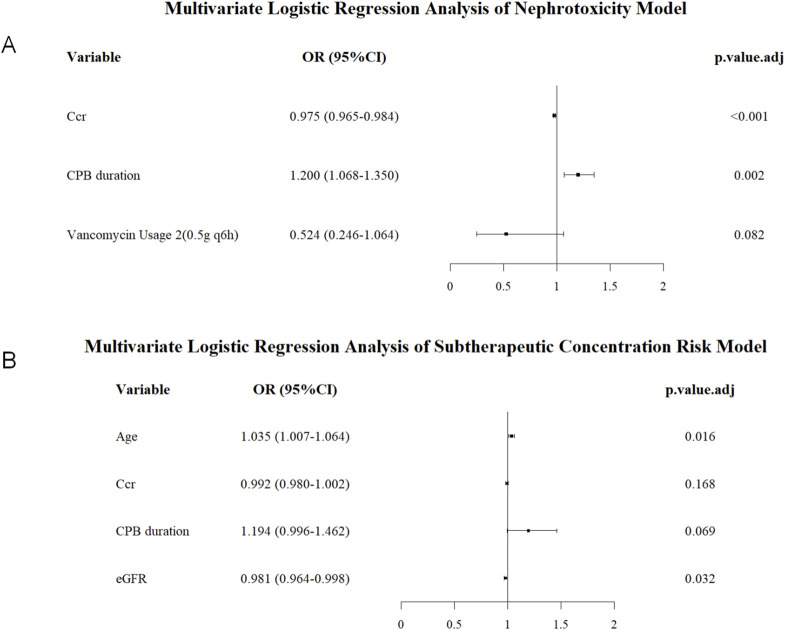
Forest plot of multivariable logistic regression analysis. **(A)** Nephrotoxicity model, **(B)** Subtherapeutic concentration risk model.

This study incorporated the predictor variables screened by LASSO regression into multivariate logistic regression analysis using bidirectional stepwise regression (forward-backward method, AIC) and constructed forest plots (Figure 3).

The nephrotoxicity risk model (AIC = 423.3) included 3 independent predictors: For each 1-h increase in cardiopulmonary bypass duration, the risk of exceeding vancomycin concentration significantly increased by 20.0% (β = 0.182, OR = 1.200, 95%CI 1.068–1.353, P = 0.002). For each 1 mL/min increase in CCr, the risk decreased by 2.5% (β = −0.025, OR = 0.975, 95%CI 0.965–0.984, P < 0.001). The second vancomycin dosing regimen (0.5 g q6h) showed a trend towards a protective effect compared to the conventional regimen (β = −0.646, OR = 0.524, 95%CI 0.246–1.064, P = 0.082).

The subtherapeutic concentration risk model (AIC = 246.0) included 4 independent predictors: For each 1-year increase in age, the risk of subtherapeutic concentration significantly decreased by 3.5% (β = 0.034, OR = 1.035, 95%CI 1.007–1.064, P = 0.016). For each 1 mL/min/1.73 m^2^ increase in eGFR, the risk increased by 1.9% (β = −0.019, OR = 0.981, 95%CI 0.964–0.998, P = 0.032). CPB duration was a borderline significant risk factor (β = 0.177, OR = 1.194, 95%CI 0.996–1.462, P = 0.069). CCr was not statistically significant (β = −0.008, P = 0.168).

**FIGURE 4:**
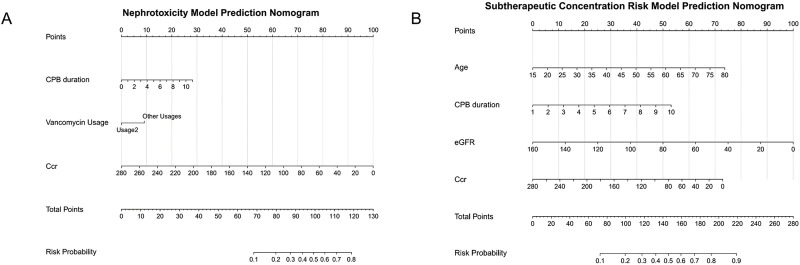
Nomogram for predicting vancomycin concentration-related risks. **(A)** Nephrotoxicity model, **(B)** Subtherapeutic concentration risk model.

Nomogram models visualizing vancomycin plasma concentration predictions based on the above variables were plotted for each group (Figure 4). The renal injury risk model (Figure 4A) employed a three-tier scoring system wherein CPB duration (0–10 h) was linearly scaled with 2-h intervals (e.g., 4 h corresponded to 10 points). The second vancomycin dosing regimen significantly mitigated risk scores compared to alternative protocols. CCr featured reverse scaling, with values declining from 280 mL/min to 0 mL/min corresponding to progressive score escalation from 0 to 100 points. The low-concentration risk model (Figure 4B) incorporated a four-dimensional scoring framework: 1) Age (15–80 years) demonstrated inverse correlation with points (e.g., 50 years yielded 40 points), 2) CPB duration (1–10 h) contributed proportionally increasing points, 3–4) Both eGFR (0–160 mL/min/1.73 m^2^) and CCr (0–280 mL/min) employed positive scaling (higher values corresponding to elevated scores, e.g., eGFR = 80 conferred 50 points).

Nomogram construction provided an intuitive risk quantification tool, enabling clinicians to derive quantitative risk predictions through parametric alignment. This methodology delivers immediate decision support for vancomycin dosing by translating patient-specific parameters into visualizable risk stratification.

### 3.5 Development and performance of the model

**FIGURE 5:**
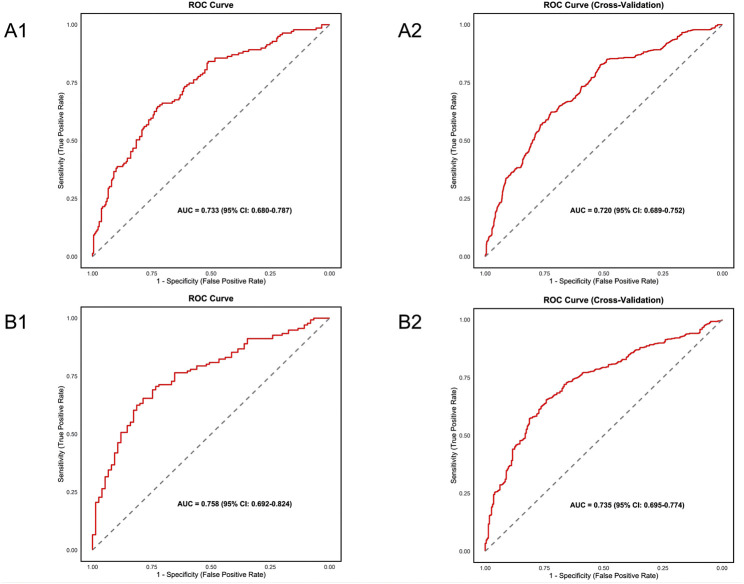
Comparison of original and 10-fold cross-validated ROC curves for the nephrotoxicity and subtherapeutic concentration risk models. **(A1)** Original ROC curve of the nephrotoxicity risk model. **(A2)** 10-fold cross-validated ROC curve of the nephrotoxicity risk model. **(B1)** Original ROC curve of the subtherapeutic concentration risk model. **(B2)** 10-fold cross-validated ROC curve of the subtherapeutic concentration risk model. ROC: Receiver Operating Characteristic.

This study rigorously evaluated the nomogram model’s performance through a multi-dimensional approach (Figure 5). The ROC curve analysis revealed an AUC of 0.733 (95%CI 0.680–0.787) for the nephrotoxicity risk group, stabilizing at 0.720 (95%CI 0.689–0.752) after 10-fold cross-validation. The subtherapeutic concentration risk group model showed better discrimination with an AUC of 0.758 (95%CI 0.692–0.824), and a cross-validated AUC of 0.735 (95%CI 0.695–0.774). Calibration was confirmed via Bootstrap (n = 1,000) and Hosmer-Lemeshow tests, showing high consistency between predicted and actual probabilities in both the nephrotoxicity group (χ^2^ = 9.50, df = 8, P = 0.302) and the subtherapeutic concentration group (χ^2^ = 9.11, df = 8, P = 0.333) (both P > 0.05).

**FIGURE 6:**
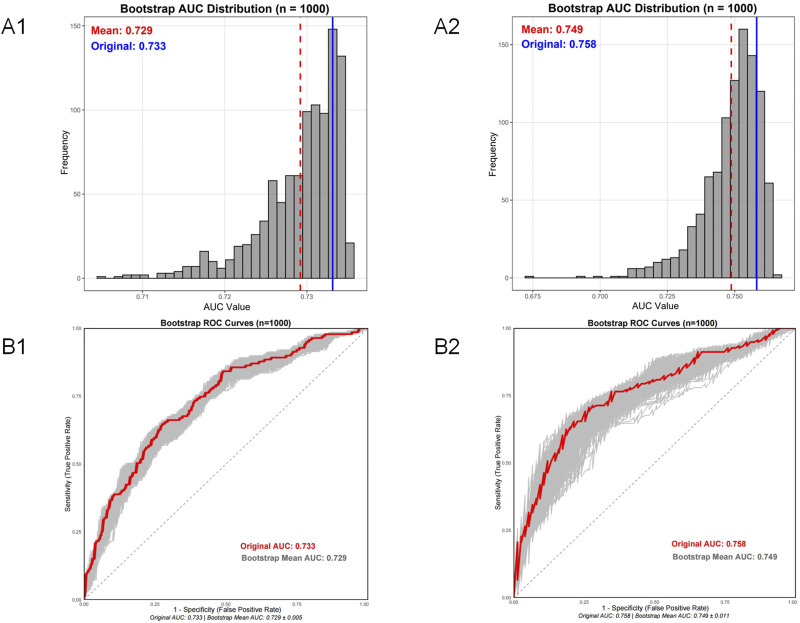
Internal validation of the nephrotoxicity and subtherapeutic concentration risk models using bootstrap resampling. **(A1)** Distributions of the bootstrap Area Under the Curve (AUC) estimates for the nephrotoxicity risk model from 1,000 resamples. **(A2)** Distribution of bootstrap AUC estimates for the subtherapeutic concentration risk model from 1,000 resamples. **(B1)** Comparison of original and bootstrap-derived ROC curves for the nephrotoxicity risk model. **(B2)** Comparison of original and bootstrap-derived ROC curves for the subtherapeutic concentration risk model.

To further assess the model’s robustness and potential for overfitting, an internal validation was performed using the bootstrap method with 1,000 resamples. The results of the bootstrap validation are summarized in Figure 6. The bootstrap-validated AUC for the nephrotoxicity risk model was 0.729 (95% CI: 0.716–0.735), and for the subtherapeutic concentration risk model, it was 0.749 (95% CI: 0.721–0.762). The minimal optimism and the narrow confidence intervals indicated excellent model stability.

**FIGURE 7:**
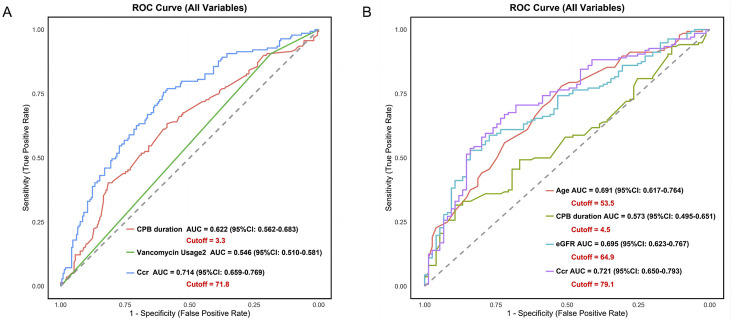
Univariate ROC Curves Based on Vancomycin Serum Concentration-Related Risk Prediction Model. **(A)** Nephrotoxicity model, **(B)** Subtherapeutic concentration risk model. ROC, Receiver Operating Characteristic.

To explore the sources of model advantages and assess key variables’ predictive abilities, univariate ROC curve analyses were performed on the predictors in both groups (Figure 7), with optimal decision thresholds for continuous variables determined by the Youden index. In the nephrotoxicity group, Ccr had the best discrimination (AUC = 0.714, 95%CI 0.659–0.769), with a cut-off of 71.8 mL/min as a key warning threshold. CPB duration was a secondary predictor (AUC = 0.622, 95%CI 0.562–0.683), indicating a positive risk effect with a risk threshold of 3.3 h. In the subtherapeutic concentration group, Ccr remained the most predictive (AUC = 0.721, 95%CI 0.650–0.793), with a cut-off of 79.1 mL/min effectively indicating suboptimal concentrations. The combined effects of age (AUC = 0.691, 95%CI 0.617–0.764) and eGFR (AUC = 0.695, 95%CI 0.623–0.767) further enhanced predictions, with cut-offs at 53.5 years and 64.9 mL/min, respectively. Ccr demonstrated the strongest predictive power in both models (AUC>0.710), with its dual-threshold system (71.8 mL/min and 79.1 mL/min) forming the core basis for vancomycin individualized dosing.

In the univariate analysis, creatinine clearance (CCr) demonstrated strong discriminative capacity for both risk models: nephrotoxicity risk (AUC = 0.714, 95%CI 0.659–0.769) and subtherapeutic concentration risk (AUC = 0.721, 95%CI 0.650–0.793). In the multivariate models, CCr was identified as a significant independent predictor for nephrotoxicity risk (β = −0.025, OR = 0.975, 95%CI 0.965–0.984, P < 0.001) but not for subtherapeutic concentration risk (β = −0.008, P = 0.168).

**FIGURE 8:**
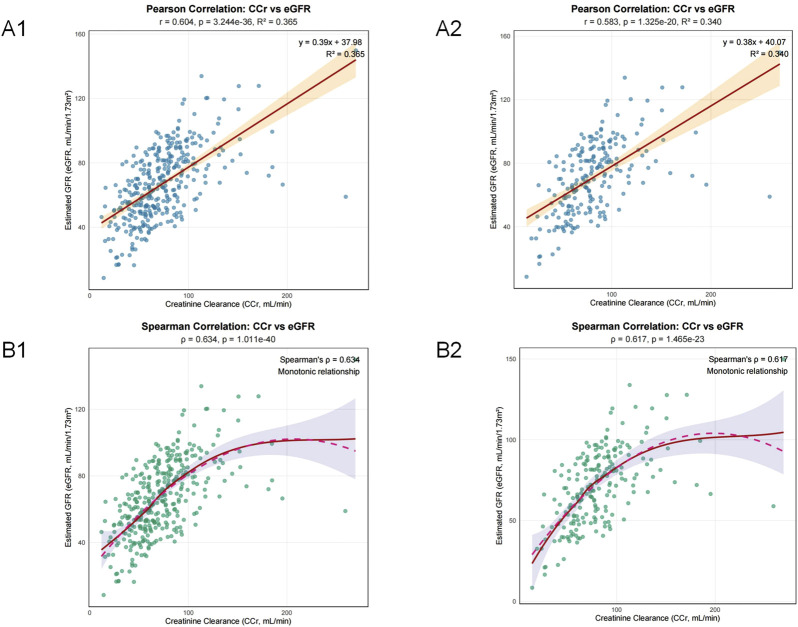
Assessment of the relationship between Creatinine Clearance (CCr) and estimated Glomerular Filtration Rate (eGFR). **(A1)** Pearson correlation analysis with linear regression fit for the nephrotoxicity risk model. **(A2)** Pearson correlation analysis with linear regression fit for the subtherapeutic concentration risk model. **(B1)** Spearman correlation analysis with non-parametric smoothing for the nephrotoxicity risk model. **(B2)** Spearman correlation analysis with non-parametric smoothing for the subtherapeutic concentration risk model.

However, partial predictive information overlap between Ccr and eGFR likely diluted Ccr’s statistical significance in multivariate modeling. To quantify this overlap, we performed correlation analysis between CCr and eGFR in both the nephrotoxicity and subtherapeutic concentration risk groups. In the nephrotoxicity model (n = 350), Pearson correlation coefficient (r) was 0.604 (R^2^ = 0.365), and Spearman’s ρ (rho) was 0.634. In the subtherapeutic concentration risk model (n = 211), Pearson r was 0.583 (R^2^ = 0.340) and Spearman’s ρ was 0.617 (Figure 8). This indicates that approximately 36.5% and 34.0% of the variance in CCr and eGFR are shared in the nephrotoxicity and subtherapeutic concentration risk groups, respectively, demonstrating a substantial information overlap. This attenuation is mechanistically expected when including biologically linked predictors and does not invalidate Ccr’s discriminative capacity.

**FIGURE 9:**
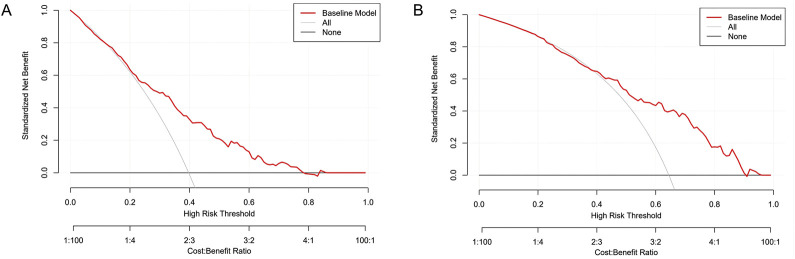
Decision Curve Analysis (DCA) Curves of Vancomycin Serum Concentration-Related Risk Prediction Model. **(A)** Nephrotoxicity model, **(B)** Subtherapeutic concentration risk model.

DCA further validated clinical applicability (Figure 9). For the nephrotoxicity model, when the high-risk threshold exceeded 10% (corresponding to cost-benefit ratios of 1:100 to 1:4), the baseline model (red solid line) showed higher net benefits than “treat all” (gray dashed line) and “treat none” (black solid line). In the core clinical decision area (cost-benefit ratios 1:4 to 1:100), net benefits increased by 0.15–0.30, suggesting 15–30 unnecessary nephrotoxicity interventions per 100 patients could be avoided. For the subtherapeutic concentration model, the baseline model maintained significant advantages across a broader threshold range (10%–80%), especially in the 20%–40% high-risk interval (cost-benefit ratios 2:3 to 3:2), with net benefit increases reaching 0.20–0.35. Even when clinicians had low tolerance for underdosing (cost-benefit ratio >3:2), the model still provided better net benefits. Both models demonstrated clear clinical value in their target risk ranges: the nephrotoxicity model excelled at avoiding overtreatment, while the subtherapeutic concentration model effectively prevented underdosing, together optimizing vancomycin individualized dosing decisions.

## 4 Discussion

Traditional therapeutic drug monitoring (TDM) for vancomycin aims to maintain concentrations within the therapeutic window to ensure efficacy and mitigate adverse effects (e.g., nephrotoxicity, ototoxicity). However, conventional TDM is limited by delayed specimen collection and suboptimal target attainment rates. To address these drawbacks, this study shifts from reactive post-administration monitoring to proactive risk prediction, developing a predictive model to identify key factors influencing vancomycin concentrations and screen high-risk patients. This approach enables early intervention, offering a distinct advantage over traditional TDM which only adjusts dosages after blood concentration testing.

Previous study has reported the derivation and validation of risk prediction models for vancomycin-associated acute kidney injury (AKI). But it only addresses the daily dose of vancomycin, and specifies the exact dosage and administration (Xu et al., 2020). However, this study explored potential predisposing factors for vancomycin-associated AKI using univariate and multivariate logistic regression. In contrast, our study specifically addresses this high-risk subgroup by constructing a dual-risk prediction model (nephrotoxicity/subtherapeutic concentration) via LASSO-logistic regression, a method that precisely screens key predictors (CPB duration, Ccr, eGFR, age) while avoiding overfitting. Validated through rigorous methods, this interpretable model directly links these clinically accessible factors to vancomycin concentration risks, laying the groundwork for personalized postoperative dosing and proactive nephrotoxicity risk warning—core advances that enhance clinical translatability.

Research on CPB’s effect on vancomycin concentrations primarily focuses on the intraoperative period during cardiac surgery. One study (Wang et al., 2023) found that CPB duration was a risk factor for vancomycin concentrations <15 mg/L at surgical closure in adult cardiac surgery patients, with patients experiencing CPB >4 h having significantly lower rates of achieving target concentrations at CPB cessation and closure. Conversely, another study (Cotogni et al., 2013) suggested that CPB did not significantly alter the intraoperative pharmacokinetics (PK) of vancomycin in patients undergoing cardiac surgery. Similar researchs (Ashkenazi-Hoffnung et al., 2024; Moffett et al., 2019) involving pediatric populations identified higher pre-operative creatinine and shorter CPB duration as predictors of supratherapeutic vancomycin concentrations. Our study found that in the renal injury prediction model: CPB duration was significantly associated with increased vancomycin concentrations, indicating that each 1-h increase in CPB time raised the risk of elevated concentrations by 20%.

This phenomenon is driven by CPB-induced physiological perturbations including hemodilution, blood component alterations, and systemic inflammatory responses (Barry et al., 2015; Mets, 2000), which exacerbate with prolonged bypass duration and disrupt vancomyci’s distribution and metabolism. Additionally, CPB impairs renal hemodynamics, reducing renal blood flow and glomerular filtration rate (Holley et al., 1982). Together, these effects diminish vancomycin excretion, leading to drug accumulation and elevated serum concentrations. For patients with CPB duration >3.3 h (our validated threshold), we recommend reduced postoperative vancomycin dosing and intensified TDM to mitigate nephrotoxicity risk.

Vancomycin is primarily renally eliminated, with its clearance closely linked to renal function (Giuliano et al., 2010; Rybak et al., 2009; Rybak et al., 2020a), consistent with studies showing a strong negative correlation between trough concentrations (Cmin) and Ccr. Patients with augmented renal clearance (Ccr≥130 mL/min) often have subtherapeutic Cmin (<10 mg/L), while those with Ccr <30 mL/min face nephrotoxicity risk from supratherapeutic levels (>20 mg/L) (Barriere et al., 2014; Claus et al., 2013; Sime et al., 2015). In our post-CPB cohort, lower Ccr correlated with higher nephrotoxicity risk, and higher eGFR with increased subtherapeutic concentration risk, findings aligning with prior research (Pan et al., 2020; Turner et al., 2018) and underscoring the need for postoperative dynamic renal function monitoring and targeted TDM to guide dosing adjustments.

We integrated three renal function indicators (SCr, Ccr, eGFR) into our prediction model to comprehensively assess vancomycin clearance in post-CPB patients. SCr provided an accessible foundational measure, while eGFR, recognized as the gold standard for renal function assessment (Levey et al., 2020; Soares et al., 2009), and Ccr, a practical surrogate derived via the Cockcroft-Gault formula (Ferreira et al., 2021; Ikegawa et al., 2018), supplemented complementary information to offset individual indicator limitations and enhance prediction accuracy. This multi-indicator approach, tailored to the dynamic renal function fluctuations post-CPB, ensured robust capture of vancomycin clearance variability, directly supporting the model’s clinical utility for personalized dosing guidance.

Age is a key determinant of vancomycin concentrations in adults, with declining GFR and reduced muscle mass in elderly patients (≥60 years) impairing drug clearance and leading to 1.5–2 times higher trough levels than younger adults (Xi et al., 2024; Yahav et al., 2019). Studies comparing trough levels in elderly (≥65 years) and younger patients confirm that uniform dosing leads to higher concentrations in the elderly. In our study’s low-concentration risk prediction group, patient age was an independent risk factor; the included population was aged >18 years. While the literature typically identifies ages >60 or 65 years as associated with higher trough levels, our study identified a cutoff of 53.5 years. Although slightly different, both findings indicate that older age correlates with a tendency for higher concentrations. Clinically, for post-CPB cardiac surgery patients, vancomycin dosing should be stratified by age. For elderly patients, particular emphasis should be placed on renal function assessment and reducing initial doses to improve target attainment within the therapeutic window.

The integration of serum creatinine (SCr), creatinine clearance (CCr), and estimated glomerular filtration rate (eGFR) provides a complementary assessment of renal function; however, their intrinsic physiological correlation requires nuanced interpretation within a multivariate framework. While CCr demonstrated strong discriminative capacity for subtherapeutic vancomycin concentrations in univariate analysis (AUC = 0.721), its statistical significance attenuated in the multivariate model after adjusting for competing predictors (P = 0.168). Notably, variance inflation factors (VIF<2) confirmed the absence of severe multicollinearity. Further correlation analysis revealed a substantial informational overlap between CCr and eGFR: in the subtherapeutic concentration risk group, the Pearson correlation coefficient was 0.583 (R^2^ = 0.340), indicating that approximately 34.0% of their variance is shared. This inherent redundancy likely diluted the independent statistical contribution of CCr in the multivariate model, whereas eGFR retained significance (β = −0.019, P = 0.032). This attenuation is mechanistically expected when including biologically linked variables and does not negate the clinical utility of CCr. Its high univariate discriminatory accuracy and the derived operational threshold (79.1 mL/min) remain directly clinically actionable, affirming CCr’s role as a practical and valuable surrogate, particularly in the acute postoperative phase when eGFR may not be readily available.

The clinical utility of our model is demonstrated by its effective integration of key risk factors, including age, into a practical decision-making framework. Using the nomogram scoring system and thresholds optimized by the Youden index, we established clear clinical pathways for vancomycin dosing in post-CPB patients. Specifically, clinicians can promptly estimate a patient’s probability of falling into a specific risk stratum by inputting their postoperative CPB duration, age, and immediate renal function indicators (CCr/eGFR) into the nomogram. Based on this stratification, patients at high risk of nephrotoxicity (e.g., CPB duration >3.3 h and CCr <71.8 mL/min) are at risk of excessive vancomycin concentrations according to our study results, so we tend to adopt a conservative initial dose of 0.5 g q8h or a lower daily dose, coupled with earlier therapeutic drug monitoring (TDM); while those at high risk of subtherapeutic concentrations (e.g., age <53.5 years and eGFR >64.9 mL/min) mean an increased risk of insufficient vancomycin concentrations, for whom we tend to use the 0.5 g q6h regimen, along with timely TDM verification. This facilitates more individualized dosing and monitoring intensity from the very beginning of therapy, with the potential to improve patient outcomes starting from the treatment initiation point.

Our study has several limitations. First, it is a single - center study with a limited sample size and lacks external validation. Second, as a retrospective study, it relies on pre-collected data, which may introduce patient selection bias. Third, despite the strong predictive power of machine learning models, there’s still an overfitting risk due to the limited sample size. Fourth, the model’s discriminative performance, reflected by ROC-AUC values of 0.733 (nephrotoxicity risk) and 0.758 (subtherapeutic concentration risk), falls within the “acceptable” range for clinical prediction models but does not reach “excellent” discrimination (AUC >0.80). Fifth, although a protective trend was observed for “Vancomycin Regimen 2 (0.5 g q6h)” in the nephrotoxicity prediction model (P = 0.082), this result requires cautious interpretation. The q6h dosing interval may help maintain stable vancomycin concentrations, potentially reducing nephrotoxicity risk associated with concentration fluctuations or prolonged supratherapeutic exposure-consistent with the pharmacokinetic principle that stable levels are critical for balancing efficacy and safety. However, this trend did not reach the conventional threshold for statistical significance (α = 0.05), and the limited sample size may have compromised statistical power, further constraining the reliability of this observation. In the future, we will expand the sample size and include data from other centers for external validation to enhance the model’s performance and generalizability. Meanwhile, we’ll combine the model with other machine learning or deep learning techniques to boost its predictive power and practicality.

## 5 Conclusion

This study investigated risk factors associated with vancomycin exposure in post-cardiopulmonary bypass (CPB) cardiac surgery patients, constructing a dual-risk prediction model via LASSO regression. Key findings revealed that lower creatinine clearance rate (CCr) and longer CPB duration predict increased nephrotoxicity risk, while younger age and higher estimated glomerular filtration rate (eGFR) were linked to an elevated risk of subtherapeutic concentrations. The model exhibited acceptable discriminative ability, and demonstrated good calibration and superior net clinical benefit through decision curve analysis (DCA). Despite the moderate discriminative performance, this model provides a practical tool for early identification of high-risk patients, enabling timely personalized dosing interventions to optimize vancomycin therapeutic outcomes while mitigating nephrotoxicity in the unique post-CPB clinical context.

## Data Availability

The original contributions presented in the study are included in the article/supplementary material, further inquiries can be directed to the corresponding authors.
